# Reports on Cities, Towns, and Districts.—Teignmouth

**Published:** 1857-10

**Authors:** W. C. Lake

**Affiliations:** Surgeon to the Teignmouth Dispensary.


### SANITARY AND SOCIAL SCIENCE.

### REPORTS ON CITIES, TOWNS, & DISTRICTS.

### REPORT ON THE SANITARY STATE OF TEIGNMOUTH.

By W. C. LAKE, M.R.C.S., M.B.M.S., Surgeon to the Teignmouth Dispensary.

TEIGNMOUTH is situated in lat. 50° 32′ N.; long. 3° 29′ W., at the mouth of the estuary of the Teign, and at the foot of a range of hills which extend nearly due W. and E. along its bank, reaching their highest elevation to the north of the town in the heathland of Haldon, and terminating in a precipitous line of cliffs on the sea coast; the declivity of the range being moulded into coombes or valleys opening out upon the river, and fronting on the opposite bank a range similar in character but of lower elevation. The sea coast itself trends in the direction of Dawlish N.N.E. The geological formation of the district is the new red sandstone, capped, as at Haldon, by the green sandstone.

Into the space occupied by the town open two valleys; that of Coombe, containing a stream of water of the same name, at its western extremity; that of Brimley, in which the Thame rivulet runs, into its eastern part. The rising ground between the two forms part of the general declivity. To the east of Brimley the ground rises towards the cliffs, interrupted only by another shallower depression. The mouth of Brimley itself opens out upon a large V-shaped flat of shingly sand and earth, which runs across the entrance into the estuary from its northern nearly to its southern side, and which, on the part fronting the sea, has been turfed, and forms an extensive promenade, called the Den. The town is built principally on the remaining portion of this flat, and on the rising ground between the vales, partly on the sides of the valleys themselves.

The width of the estuary opposite the town is about 650 yards. At low water several banks are exposed in its course. Those in the neighbourhood of the town are composed entirely of sand and shingle. The one nearest the town, of large size, and which begins to be uncovered soon after high water, is always fringed with fresh seaweed, and gives to this part of the estuary rather the character of an involution of the sea-shore.

No trustworthy observations, that I am aware of, had been made of the meteorology of Teignmouth previously to the spring of 1854; a sufficiency of time, therefore, has not elapsed to enable one to deduce its mean elements from those that have been taken since that date. The following table contains the means of the several observations during the two years commencing with April 1854, and closing with March 1856. As this has been in many respects an exceptional period, I have subjoined, as far as I was able, similar tables, taken for the same time, and the means for Exeter and Torquay, using the Registrar General's Quarterly Report. (See next page.)

In any respect these tables have a merely approximative value.

The several parts of the town present more features of difference, with regard to climatic character, than might have been expected from its comparatively limited size. The part situated on the flat, and which contains a principal portion of the better class of houses for residents, and almost all the lodging houses, faces, generally speaking, S.E., and is a good deal exposed to easterly winds, though forming a delightful place of summer residence. The part on the rising ground between the vales faces nearly S., has the advantage of a more elevated situation than the first part, and is more sheltered by the hills rising to the north of the town, and which, at a distance of two miles from it, attain an elevation of from 500 to 600 feet. It is less exposed to the east wind, and is mostly occupied by houses belonging to the poorer classes, though containing also some of the better class of houses, and a few of the lodging houses. It is in this part of the town that the meteorological observations have been taken. The sides of the valleys, which combine the advantages of elevation to a greater or less degree, and, on one side at least, complete shelter from easterly winds, and are well fitted for a place of winter resort, are at present occupied by a comparatively small portion of the town, consisting chiefly of gentlemen's seats and villa residences, with a few of the houses of the labouring population. It is, however, in the direction of these valleys that the town is now extending.

The excavation of the South Devon Railway, which passes through the town, caused the destruction of many of the poorer class of houses. It not only threw a considerable temporary influx of labourers into the town, but since that time the poorer part of the population has seemed to exist in a higher ratio than formerly. As a consequence of this, and of the high price of land in the town, most of the unoccupied pieces of ground have been used for the erection of houses for the accommodation of the poor. These edifices are small in extent of ground surface, but make up in height and in the number of the rooms. If this plan of building were to continue, it would tend to interfere much with the due ventilation of the narrower streets, and in other ways to unfavourably affect them.

A year or two ago, a stack of model cottages, in three tiers, was built, each house being separate, and each having

### TEIGNMOUTH.

| | Jan. | Feb. | March. | April. | May. | June. | July. | Aug. | Sept. | Oct. | Nov. | Dec. |
|---|---|---|---|---|---|---|---|---|---|---|---|---|
| Mean barometer . . . . . . | 29·83 | 29·82 | 29·85 | — | — | 29·90 | 29·87 | 29·96 | 30·10 | 29·73 | 29·89 | 29·90 |
| Mean maximum temperature . | 44·1 | 42·9 | 46·6 | 55·0 | 58· 6 | 64·1 | 68·7 | 69·4 | 65·1 | 58·1 | 48·0 | 46·9 |
| Mean minimum temperature . | 37·0 | 33·8 | 36·3 | 41·1 | 44· 2 | 50·1 | 54·1 | 54·5 | 52·2 | 46·8 | 38·4 | 38·6 |
| Mean temperature . . . . . | 40·2 | 38·1 | 40·5 | 46·8 | 49· 9 | 55·2 | 59·8 | 60·2 | 58·0 | 51·5 | 43·0 | 42·8 |
| Mean humidity . . . . . . | 88 | 85 | 82 | 73 | 77 | 80 | 83 | 79 | 81 | 83 | 83 | 84 |
| Mean total rain . . . . . . | 2·2 | 2·8 | 2·3 | 0·3 | 2· 8 | 2·4 | 2·2 | 0·9 | 0·6 | 3·5 | 1·3 | 1·6 |
| Mean ozone (Schonbein) . . | 4·9 | 5·2 | 3·8 | 4·5 | 5· 3 | 4·6 | 2·8 | 2·6 | 3.1 | 4·4 | 3·6 | 5·0 |
| Mean daily range . . . . . | 7·1 | 9·1 | 10·3 | 13·9 | 14· 4 | 14·0 | 14·6 | 14·9 | 12·9 | 11·3 | 9·6 | 8·3 |

### EXETER.

| | Jan. | Feb. | March. | April. | May. | June. | July. | Aug. | Sept. | Oct. | Nov. | Dec. |
|---|---|---|---|---|---|---|---|---|---|---|---|---|
| Mean barometer . . . . . . | 29·74 | 29·78 | 29·77 | — | — | 29·82 | — | — | — | 29·63 | 29·84 | 29·82 |
| Mean maximum temperature . | 45·4 | 44·3 | 49·3 | 59·1 | 60· 3 | 65·5 | — | — | — | 59·1 | 50·4 | 47·7 |
| Mean minimum temperature . | 35·3 | 32·5 | 34·9 | 39·7 | 43· 8 | 49 8 | — | — | — | 44·8 | 36·4 | 36·3 |
| Mean temperature . . . . . | 40·1 | 38·3 | 41·0 | 48·5 | 51· 0 | 56·0 | — | — | — | 51·5 | 43·0 | 42·4 |
| Mean humidity . . . . . . | 86 | 85 | 83 | 71 | 74 | 78 | — | — | — | 84 | 84 | 85 |
| Mean total rain . . . . . . | 2·5 | 2·4 | 2·0 | 0·5 | 2· 4 | 3·0 | — | — | — | 3·2 | 1·3 | 1·9 |
| Mean daily range . . . . . | 10·1 | 11·8 | 14·4 | 19·4 | 16· 5 | 15·7 | — | — | — | 14·3 | 14·0 | 11·4 |

### TORQUAY.

| | Jan. | Feb. | March. | April. | May. | June. | July. | Aug. | Sept. | Oct. | Nov. | Dec. |
|---|---|---|---|---|---|---|---|---|---|---|---|---|
| Mean barometer . . . . . . | — | — | — | — | — | — | — | — | — | — | — | — |
| Mean maximum temperature . | 44·3 | 42·5 | 45·8 | 56·4 | 56·7 | 61·3 | — | — | — | 57·2 | 47·8 | 46·8 |
| Mean minimum temperature . | 37·4 | 36·2 | 38·1 | 42·4 | 44·8 | 48·0 | — | — | — | 47·9 | 40·2 | 39·5 |
| Mean temperature . . . . . | 41·0 | 39·1 | 41·3 | 47·9 | 49·2 | 53·2 | — | — | — | 52·3 | 43·7 | 42·9 |
| Mean humidity . . . . . . | 81 | 85 | 81 | 63? | 70? | 79 | — | — | — | 80 | 80 | 85 |
| Mean total rain . . . . . . | 1·7 | 1·6 | 1·8 | — | — | — | — | — | — | 3·1 | 2·1 | 1·8 |
| Mean daily range . . . . . | 6·9 | 6·3 | 7·7 | 14·0 | 11·9 | 13·3 | — | — | — | 9·3 | 7·6 | 7·3 |

attached every necessary convenience, and with larger rooms than in the generality of the poorer houses; the whole being well drained and situated in a high and open part of the town. If one may judge from the short period of time that has elapsed since their erection, they may be spoken of most favourably, both in regard to the comfort and the health of their occupiers.

By an Act of Parliament, obtained in 1836, the power of lighting, watching, draining, and otherwise generally improving the town, was vested in the hands of commissioners elected from amongst the ratepayers. By means of this body, streets have been widened and otherwise improved, a police has been established, and the town has been lighted with gas and supplied with water. Before this time the water supply of the inhabitants had been obtained from wells and the Coombe and Brimley brooks; it is now almost entirely supplied by means of a reservoir situate in the Coombe valley, at a higher elevation than the major part of the town, and fed by the Coombe brook, the water being taken from it near its source on the side of Haldon. A few only of the private wells remain.

The declivity on which a great part of the town is built offers great advantages for draining, while the surface water rapidly passes off; from the higher parts, owing to the great fall; from the lower, on account of the porous nature of the soil. Teignmouth cannot, however, boast of an efficient sewerage. Much has been done from time to time by the commissioners, in covering open drains and forming new ones, but most of the old drains are small and imperfectly constructed, and the increased number of houses in the streets has rendered them still further inefficient. Some of the streets would even appear to be wanting in public drains to a great extent, if not altogether; while the main sewer, consisting of the Thame rivulet, arched over in its passage through the town, and emptying itself into the river, is uncovered nearly through its whole extent between high and low-water mark, and, assisted by some smaller drains, which are similarly circumstanced, is, especially in certain winds, a most serious annoyance to all the houses in its vicinity.

A few years ago a plan for the extinction of these nuisances, and for the effectual drainage of the town, was laid before the commissioners, and met with their approval at the time. Measures have lately been taken for covering in the principal sewer as far as the low-water mark. It is to be hoped that this step will be followed by others in the same direction, and that the ratepayers will be disposed to vote a sufficient supply of money for the purpose.

When the cholera last invaded England, a temporary local Board of Health was instituted to make a house by house visitation; to examine into the existence of nuisances; and to take steps for their removal. An opportunity was thus afforded for seeing the condition of the generality of houses in their separate drainage arrangements. On the whole, this may be said to be satisfactory. Cesspools have been interdicted, and the use of pans, with syphon tubes, as far as possible enforced.

The population of Teignmouth, at the last census, was a little over 5000. It is, besides being a place of general residence, a watering-place and a sea-port. The labouring population are probably pretty equally divided amongst field labourers, artificers, and those engaged in the various handicraft employments, and seamen, fishermen, and those concerned about the shipping and the river trade. It may, I think, be doubted whether, except in certain cases, there is much absolute poverty in the town, such at least as exists in larger places. Idleness, mismanagement, and improvidence, are, I fear, more often the cause of temporary want, than real inability to acquire a sufficient maintenance by the labour of the hands.

The two parishes of East and West Teignmouth are separated by the Thame brook, which, being covered over in its passage through the town, does not form a line of demarcation recognisable by the eye; and, indeed, in a few instances, part of a house is in one parish, part in the other. The burial grounds of both parish churches, and of nearly all the dissenting places of worship, have been lately closed by an order in council, and a cemetery, prettily situated, has been formed, under the "Burials beyond the Metropolis Act," to the north, and at the distance of about three-quarters of a mile from the town.

Teignmouth has the reputation of being a very healthy place, and I believe justly, not merely from the longevity and hale old age of many of its inhabitants, but from the comparatively unfrequent occurrence of epidemics, and their usual mildness when they do occur. Small-pox has not visited the town since the autumn of 1852, and then only transiently. Scarlatina showed itself in the autumn of 1853, and cases since occurred till the spring of 1856, for the most part mild in character, a few only having a fatal termination. Of cholera three cases alone are said to have occurred, all in old women, during the epidemic of 1832.

The type which fever assumes here may be denominated gastro-bilious. It has in the main similar characteristics in children and in adults, except that in the former a tendency to remission is prominently marked; in the latter, this is less prominent and occurs less frequently. The symptoms of a normal attack are the usual symptoms of pyrexia, accompanied with some epigastric pain and tenderness, nausea, and frequently vomiting, the skin being more or less tinged with bile, and the motions dark coloured and offensive, at times almost black; the febrile symptoms lasting for a period varying between eight or ten days and two or three weeks, when the tongue cleans, and the appetite and strength return. Cases of this fever occur usually in the course of every year, more commonly amongst children. Its occurrence in separate cases seems to be more dependent on meteorological causes, or on constitutional or possibly miasmatic conditions, than on contagion. A fatal termination is very rare. Rheumatic fever is not common.

Pneumonia is not common; and idiopathic pleurisy is of great rarity. Phthisis also is by no means of common occurrence amongst the resident population.

The class of disorders to which there appears to be the greatest proclivity, are those connected with derangement of the chylopoietic viscera, the whole group of bilious and stomach complaints.

I regret that I have not yet been able to obtain any information with regard to the average yearly proportion of deaths.

The general healthiness of the town is not very easily accounted for. Its sanitary arrangements are not apparently superior to those of neighbouring towns; yet, while several of these have, during the past two or three years, been the subjects of severe epidemics, Teignmouth has either escaped altogether, or has suffered from the same complaints in a mild form. These disorders do of course at times occur of a formidable type, but certainly not commonly, nor so frequently as in some other places. The immunity from small-pox for the last four years, is no doubt partly owing to the attention paid to public vaccination, and to the tolerable readiness with which it is had recourse to by the poor. Here, at least, the Compulsory Vaccination Act has worked well, not from its compulsory clauses having been put in force, but from the people becoming habituated to regard the operation as a thing which is required to be performed, and from the knowledge of the existence of compulsory clauses affording the necessary stimulus to some, who would otherwise allow their prejudices to prevail.

Perhaps much of the salubrity of the town is owing to its position, the sea being in front, the pure air of Haldon descending on it from behind, while the interchange of easterly and westerly winds causes a continual current over the town, from the sea in the direction of the river, and from Dartmoor down the river towards the sea. Were sanitary improvements more fully carried out, and their continuance enforced, Teignmouth would probably yield to few towns of its size, in the general healthiness of its inhabitants, and its freedom from fatal disease.

---

### MISCELLANEA.

